# 

**Published:** 1893-11

**Authors:** 


					﻿'THE
Southern Medical Record
A Monthly Journal of Medicine and Surgery.
VOL. XXIII.	Atlanta, Ga., November, 1893.	NO 11.
EDITORS:
A. W. GRIGGS, M. D.	WILLIS F. WESTMORELAND, M. D.
j. mcfadden gaston, m. d. ■ louis h. jones, m. d.
DAN. H. HOWELL, M. D., Bus. Mgr.
Entered at the Postoffice at Atlanta, Ga., as Secpnd-Class Matter.
Mutual Printing Company, Publishers, 27 East Hunter St., Atlanta, Ga.
				

